# Supplementary material for: A global resource for genomic predictions of antimicrobial resistance and surveillance of Salmonella Typhi at pathogenwatch
Source: Nat Commun. 2021 May 17;12:2879. doi: 10.1038/s41467-021-23091-2 (PMC8128892; doi:10.1038/s41467-021-23091-2)
Supplement: Supplementary file 1 — Supplementary Information [file 41467_2021_23091_MOESM1_ESM.pdf]

A global resource for genomic predictions of antimicrobial resistance and surveillance of *Salmonella* Typhi at Pathogenwatch

Silvia Argimón, Corin A. Yeats, Richard J. Goater, Khalil Abudahab, Benjamin Taylor, Anthony Underwood, Leonor Sánchez-Busó, Vanessa K. Wong, Zoe A. Dyson, Satheesh Nair, Se Eun Park, Florian Marks, Andrew J. Page, Jacqueline A. Keane, Stephen Baker, Kathryn E. Holt, Gordon Dougan, and David M. Aanensen.

Supplementary Information

This file includes:

Supplementary Figures 1-8

Supplementary Tables 1-4

Supplementary References

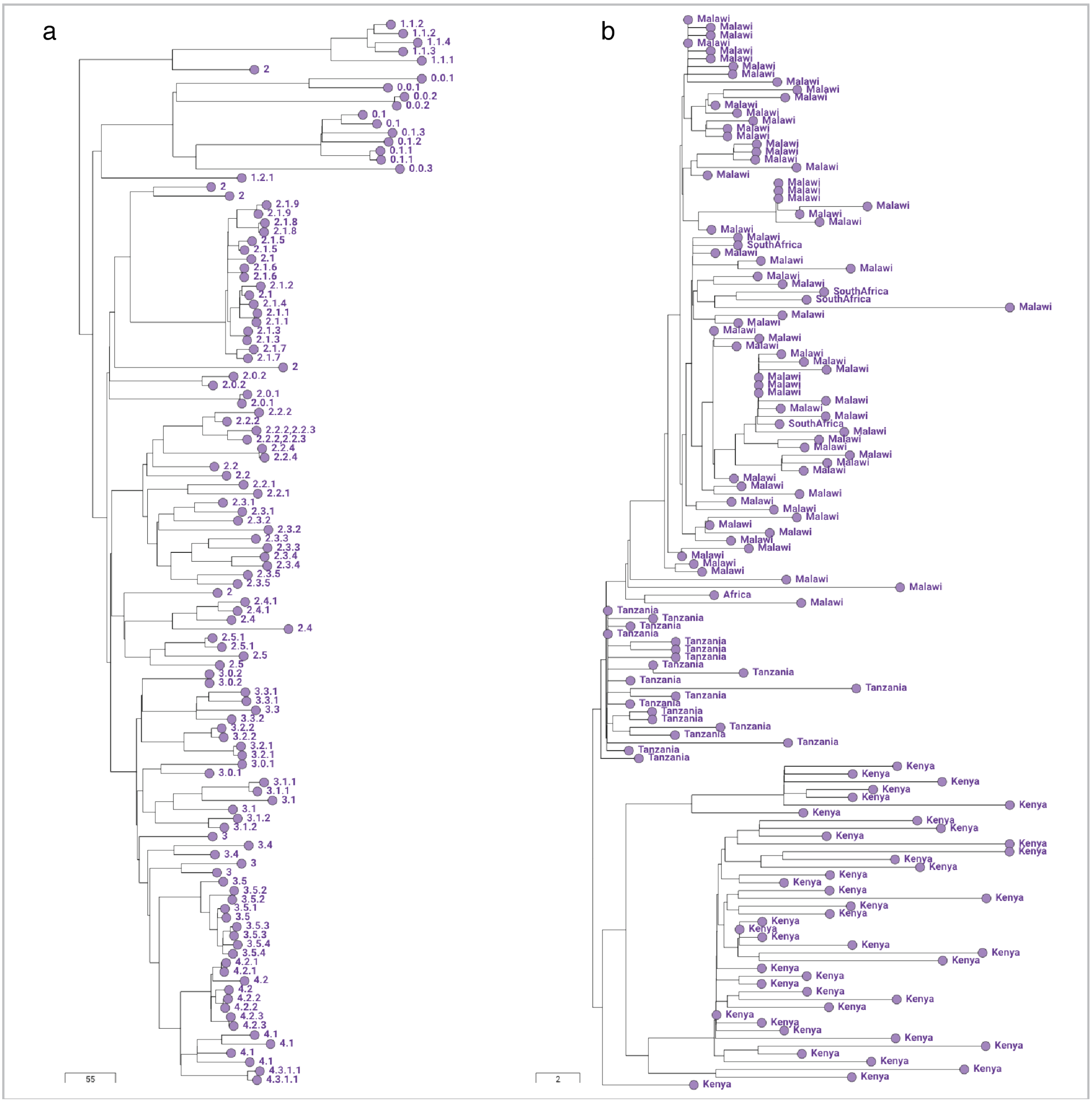

Supplementary Figure 1. a Pathogenwatch collection tree of 118 diverse *S.* Typhi genomes (dataset I) representing 62 genotypes (tree labels). **b** Pathogenwatch collection tree of 138 closely-related genomes from a clonal expansion within East Africa (dataset II). The tree nodes are labelled by country of isolation. The scale bars represent the number of SNP differences.

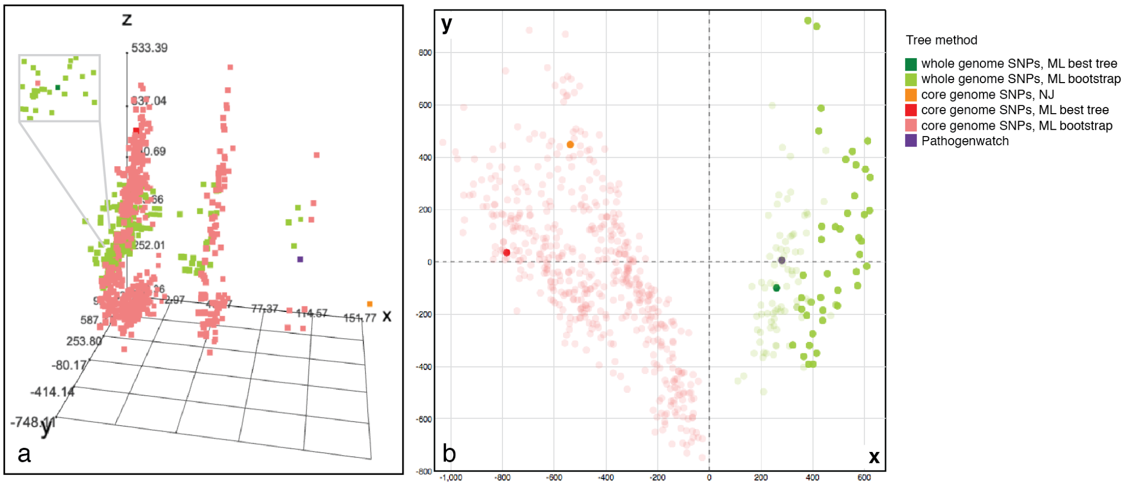

	pathogenwatch	roary_raxml	roary_NJ	mapping_raxml
pathogenwatch	0	1232.87	1103.917	261.7919
roary_raxml	126.8621	0	900.6298	1213.995
roary_NJ	67.09694	167.5112	0	1127.043
mapping_raxml	123.065	36.59235	167.544	0

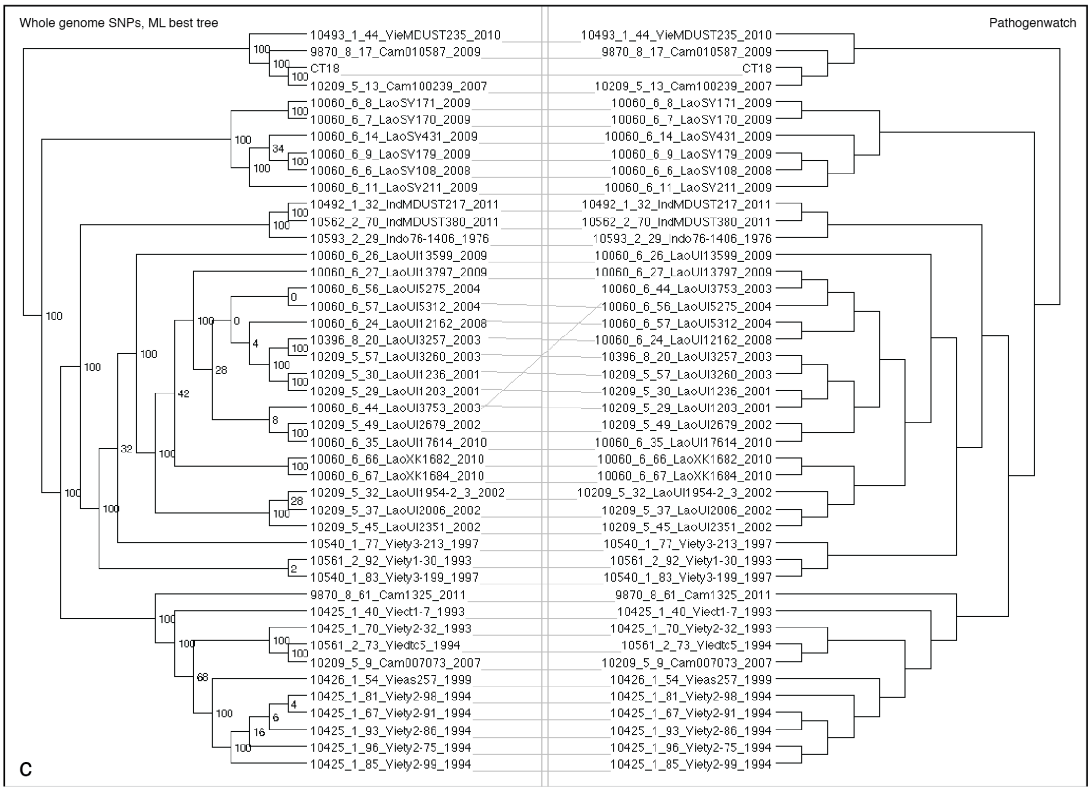

Supplementary Figure 2. a Three-dimensional multidimensional scaling (MDS) plot of pairwise tree distances from dataset I (118 genomes), and **b** Two-dimensional MDS plot of pairwise tree distances from dataset II (138 genomes), both generated with Treescape. Trees were generated with four methods: 1) Pathogenwatch (purple); 2) Maximum likelihood on 9112 SNPs identified on an alignment of 3996 concatenated core genes estimated with roary (best tree, red); 3) Neighbour joining on the same alignment in 2) (orange); and 4) Maximum likelihood on 8598 SNPs called on a CT18-guided alignment (best tree, green). Five hundred bootstrap replicates were computed for each of the methods 2 (pink) and 4 (lime green). The inset in **a** unveils the position of the best tree from method 4 (green). The matrix shows the pairwise distances between the trees from methods 1-4 for dataset I (below diagonal) and dataset II (above diagonal). **c** Tanglegram comparison of the Pathogenwatch tree of 43 genomes from subclade 3.2.1 (right) with the CT18-guided alignment tree from method 4 (left).

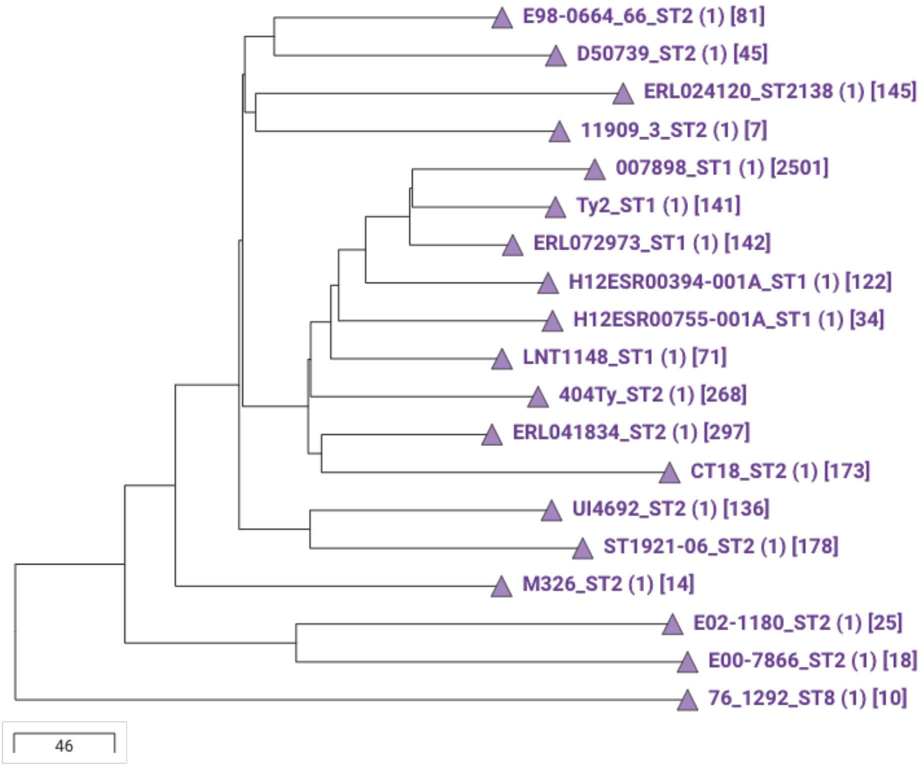

Supplementary Figure 3. The Typhi Pathogenwatch population Tree of 19 reference genomes, inferred from 2409 polymorphic sites found in 1639 of the 3916 core gene families. The numbers in parenthesis indicate the number of user genomes that have been sub-clustered with each reference in the tree. The numbers in brackets indicate the number of public genomes available on each subtree, which are also clustered with the user genomes.

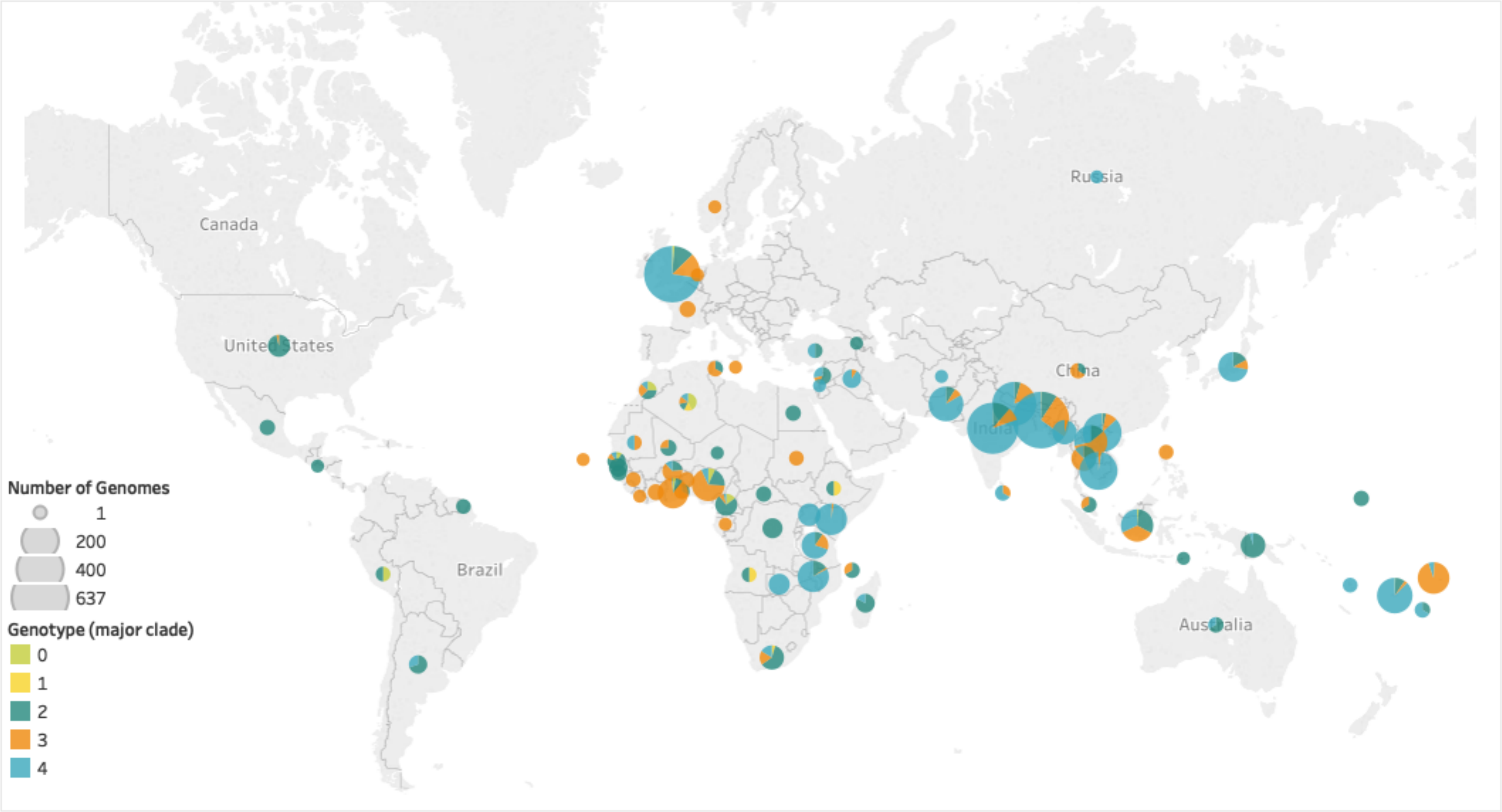

Supplementary Figure 4. Geographic distribution of public *S.* Typhi genomes available in Pathogenwatch. The size of the markers is proportional to the number of genomes in each country. The pie charts show the relative abundance of the four major clades per country. The visualization was created with Tableau Desktop v2018.3.

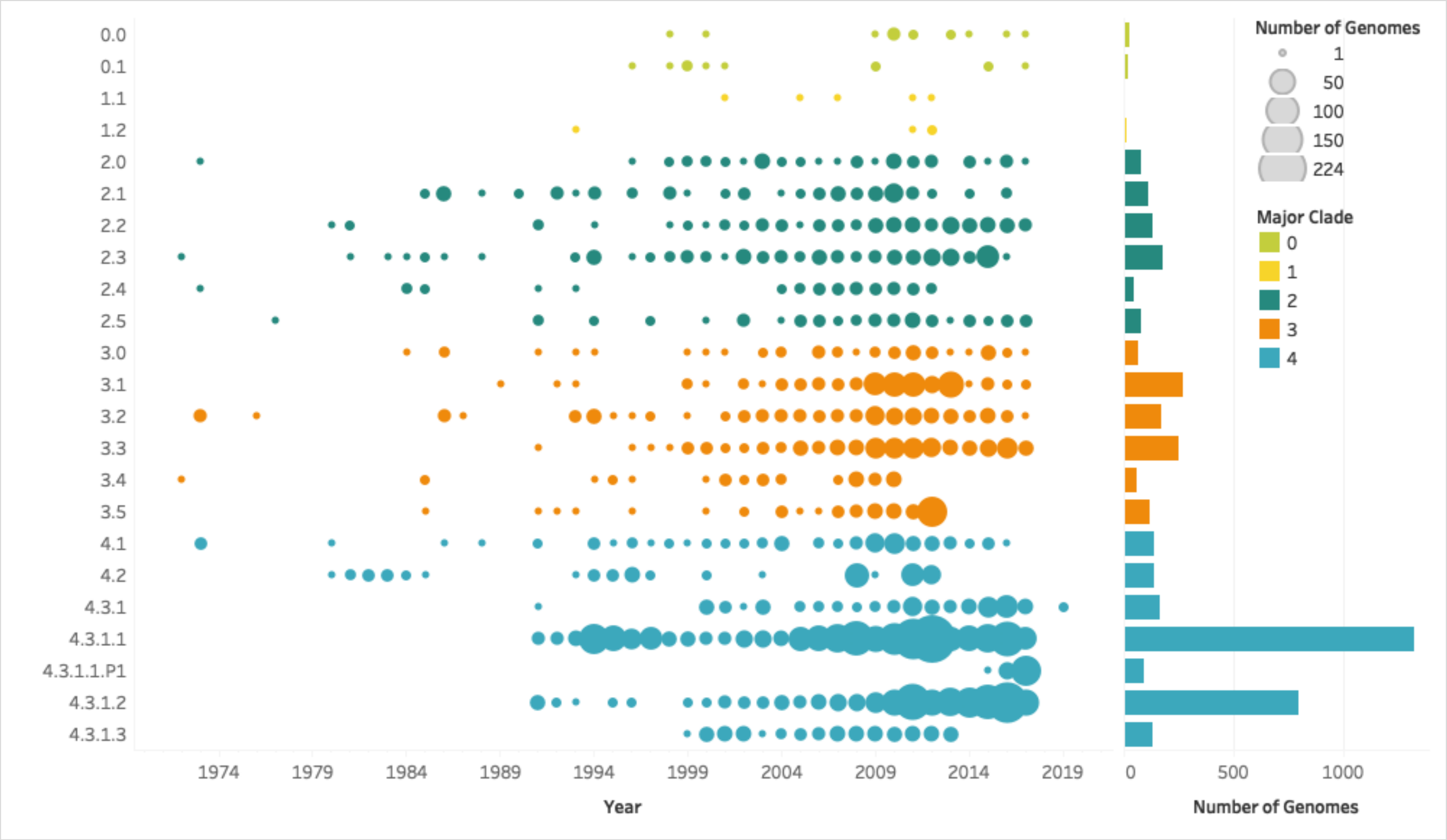

Supplementary Figure 5. Temporal distribution (left) and frequency (right) of the genotypes in the Typhi Pathogenwatch public collection of genomes. Genotypes were grouped by clade (e.g. 4.2) with the exception of 4.3, which was further discriminated into subclades. The markers on the timeline were coloured by the major clade they belong to, and their size is proportional to the number of genomes. The bar chart show the frequency of the genotypes, coloured by the major clade they belong to. Isolates collected before 1970 were excluded from the graphs. The visualization was created with Tableau Desktop v2018.3.

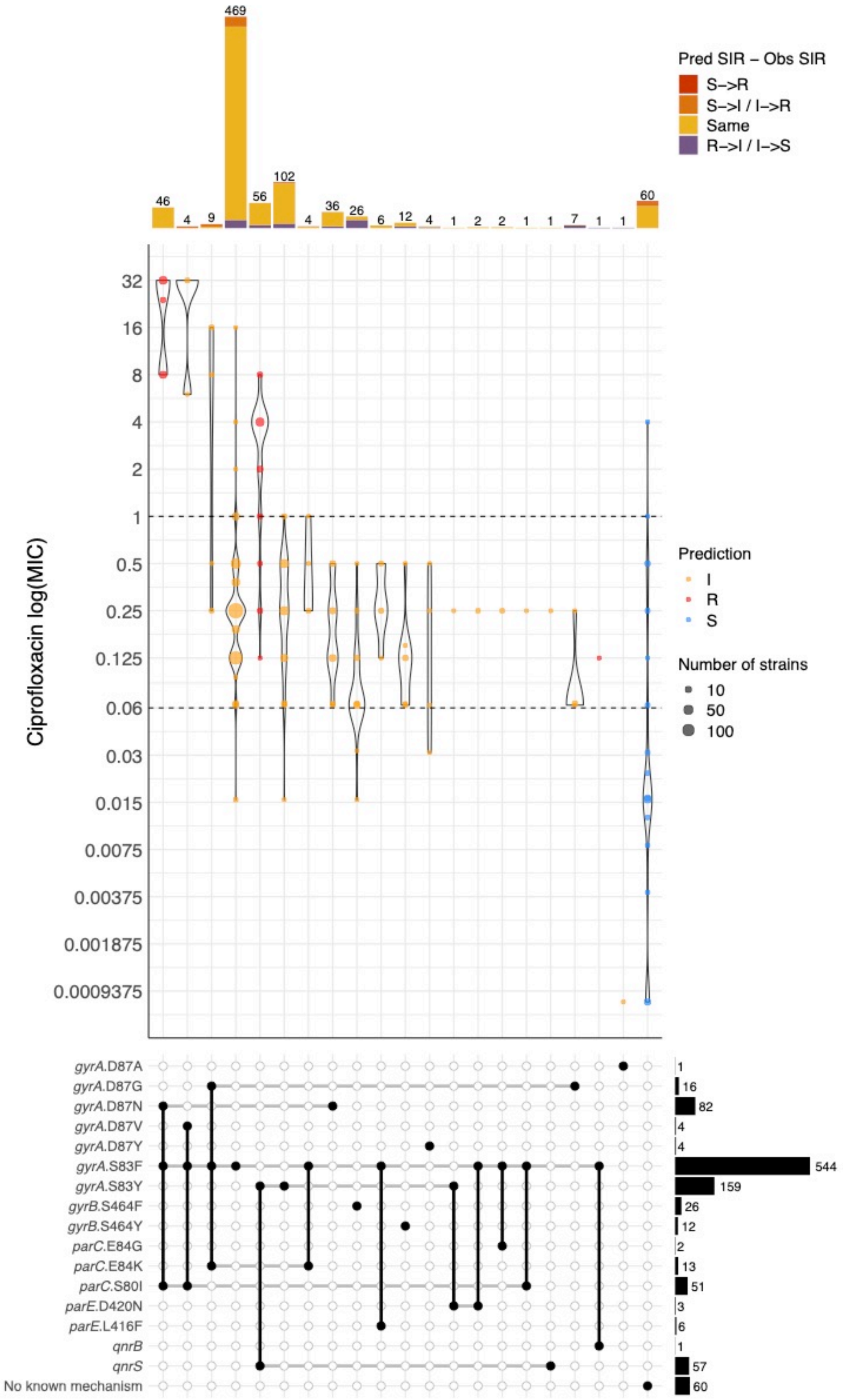

Supplementary Figure 6. Distribution of minimum inhibitory concentration (MIC) values (mg L^{-1}) for ciprofloxacin in a collection of 889 *S.* Typhi isolates with different combinations of genetic mechanisms that are known to confer resistance to this antibiotic. Dashed horizontal lines on the violin plots mark the CLSI clinical breakpoint for ciprofloxacin. Point colours inside violins represent the genotypic AMR prediction by Pathogenwatch on each combination of mechanisms. Bar plots on the top show the abundance of genomes with each combination of mechanisms. Bar colours represent the differences between the predicted and the observed SIR (e.g. red for a predicted susceptible mechanism when the observed phenotype is resistant). S = susceptible, I = intermediate, and R = resistant.

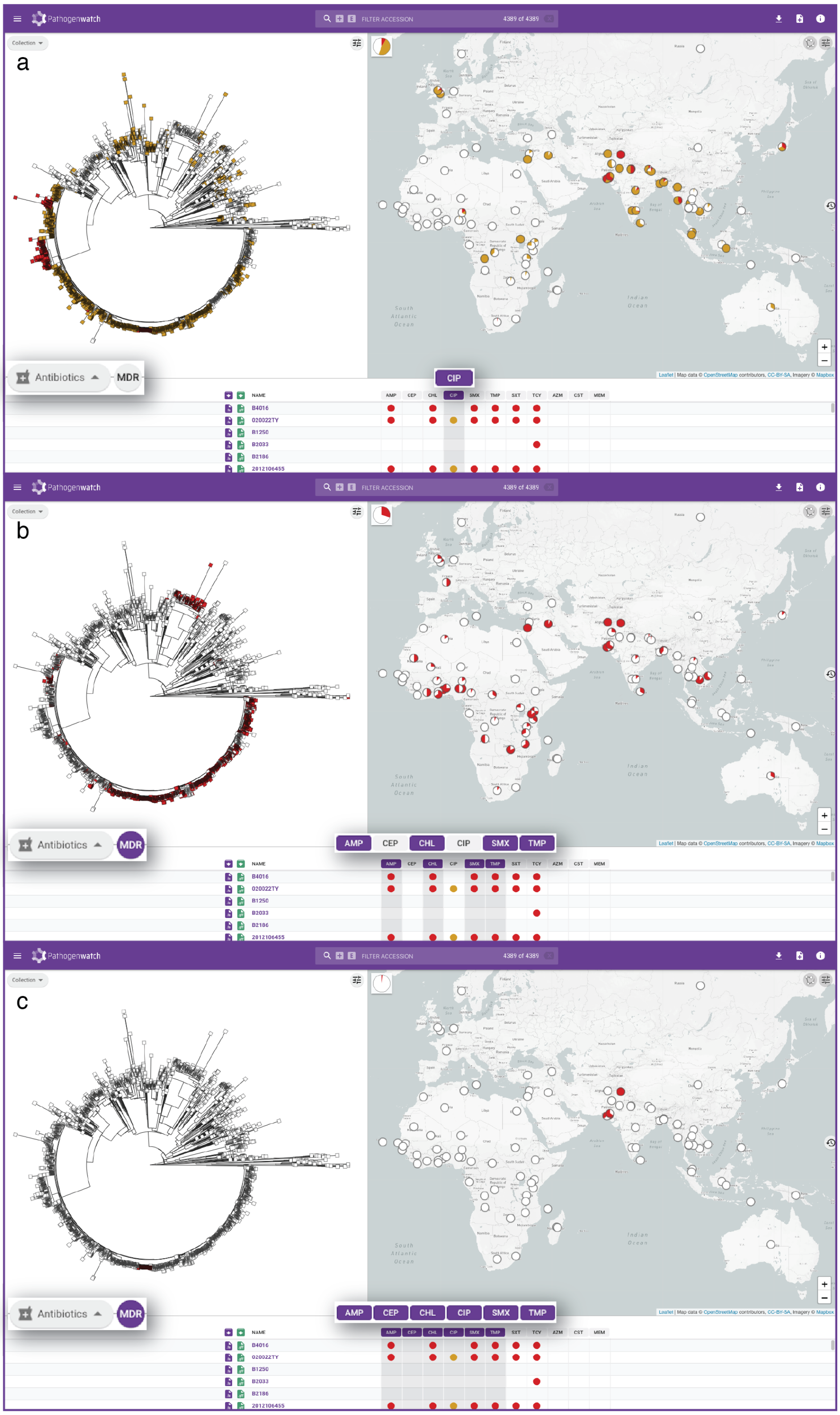

Supplementary Figure 7. Distribution of predicted (**a**) ciprofloxacin-resistant (CIP), (**b**) MDR (resistant to ampicillin, chloramphenicol, sulfamethoxazole, and trimethoprim), and (**c**) XDR (MDR with additional resistance to ciprofloxacin, and extended-spectrum cephalosporins) *S.* Typhi on the map and tree of 4389 genomes. Tree nodes are coloured red (resistant), yellow (intermediate), or white (no resistance determinants found). The map pie charts display the proportion of resistant/intermediate genomes at each location. The expandable pie chart at the top-left corner of the map view indicates the proportion of resistant/intermediate genomes for the entire set of 4389 genomes.

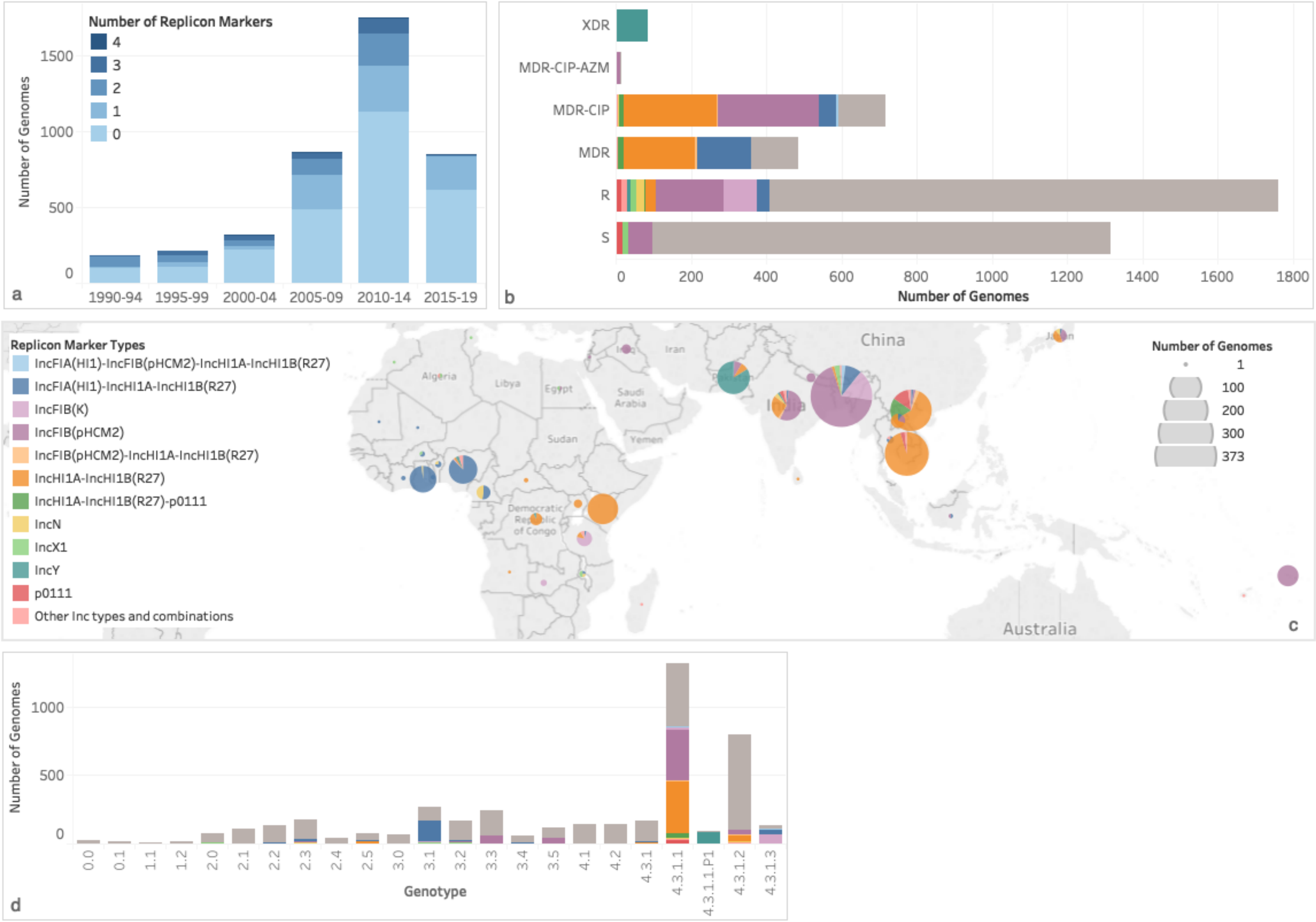

Supplementary Figure 8. Distribution of plasmid replicon marker types across the 4389 public genomes in Typhi Pathogenwatch. **a** Number of replicon markers per year interval. The genomes from isolates collected before 1990 were excluded. **b** Distribution of replicon marker types across the different predicted resistance profiles grouped as in Supplementary Figure 4. **c** Geographical distribution. **d** Distribution of replicon types across the different predicted genotypes. The visualization was created with Tableau Desktop v2018.3.

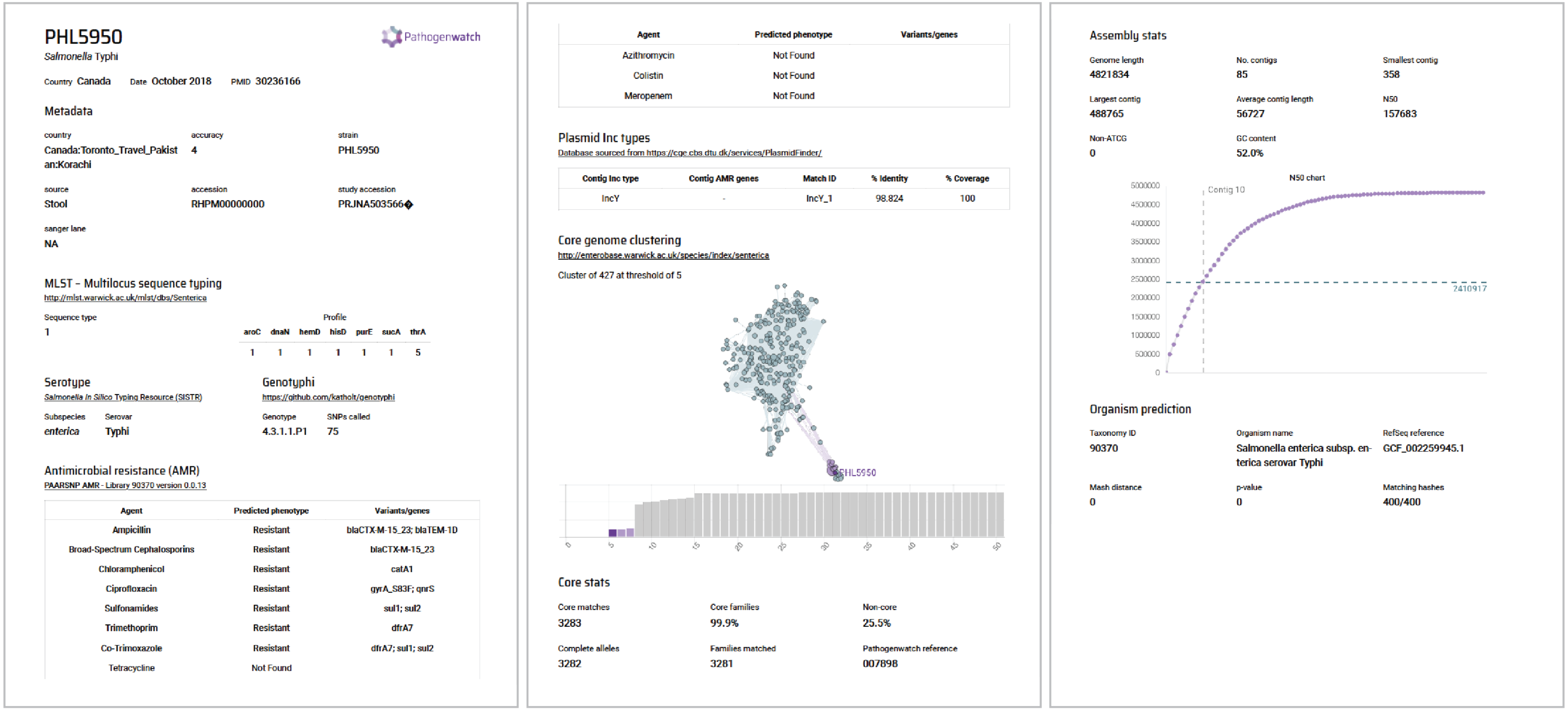

Supplementary Figure 9. Pathogenwatch genome report for assembly PHL595.

Supplementary Table 1. Collections of *S.* Typhi genomes included in Pathogenwatch at the time of writing. The availability of strain-level antimicrobial susceptibility testing (AST) data and of MIC values for ciprofloxacin is indicated.

Collection name	PMID	No. of Genomes	Location	AST performed/ data used for concordance	CIP MIC data available	Reference
Ahmad et al. (2017)	28254988	1	Malaysia	yes / no	no	(1)
Ashton et al. (2016)	27069781	513	UK (Travel multi)	no	no	(2)
Baker et al. (2015)	26411565	37	NA	no	no	(3)
Britto et al. (2018)	29684021	192	Nepal	yes / yes	no	(4)
Britto et al. (2020)	31665304	94	India	yes / yes	no	(5)
Burnsed et al. (2018)	30236166	30	USA (Travel Marshall Islands)	yes / no	no	(6)
Djeghout et al. (2018)	29616895	1	Bangladesh	yes / yes	yes	(7)
Dyson et al. (2017)	28060810	45	Thailand	no	no	(8)
Gul et al. (2017)	29051234	1	Pakistan	yes / no	no	(9)
Hendriksen et al. (2015)	25392358	22	Zambia	yes / no	no	(10)
Hendriksen et al. (2015)	25428145	2	Netherlands and Norway (Travel Philippines)	yes / yes	yes	(11)
Hooda et al. (2019)	31730615	12	Bangladesh	yes / yes	no	(12)
Ingle et al. (2019)	31513580	524	UK (Travel multi)	yes (N=173) / yes	yes	(13)
Klemm et al. (2018)	29463654	100	Pakistan	yes / yes	no	(14)
Matono et al. (2017)	29255729	93	Japan (Travel multi)	yes / yes	no	(15)
Oo et al. (2019)	31225619	39	Myanmar	yes / yes	yes	(16)
Park et al. (2018)	30504848	249	Africa multi	no	no	(17)
Pham Thanh et al. (2016)	26974227	78	Nepal	yes / yes	yes	(18)

Phoba et al. (2017)	29136410	1	DR Congo	yes/no	no	(19)
Pragasam et al. (2020)	32003431	194	India	yes (N=30) / yes	no	(20)
Rodrigues et al. (2017)	25961941	3	India	yes / no	no	(21)
Sah et al. (2019)	31872221	2	India	yes / yes	yes	(22)
Tanmoy et al. (2018)	32003431	536	Bangladesh	yes / yes	yes	(23)
Wong et al. (2015)	25961941	1822	Multi	no	no	(24)
Wong et al. (2016)	27703135	99	Multi	no	no	(25)
Wong et al. (2016)	27657909	128	Nigeria	yes / no	no	(26)

Supplementary Table 2. List of genetic AMR determinants in Typhi Pathogenwatch. Effect: R = Resistance, I = Intermediate resistance (decreased susceptibility). Genes and mutations in bold type have been demonstrated to have a role on AMR in *S.* Typhi, the remaining mechanisms are of emerging importance in Gram negative bacteria. Pathogenwatch Library 1: *S.* Typhi AMR (27), 2: ESBL (28), 3: Carbapenemases (29), 4: Colistin resistance (30)

Antibiotic class	Antibiotic (abbreviation used in Pathogenwatch)	Genetic determinants of resistance	Effect	Pathogenwatch Library (References)
Phenicols	Chloramphenicol (CHL)	***catA1***	R	1 (31)
		cmlA	R	1 (32)
Fluoroquinolones	Ciprofloxacin (CIP)	***qnrS***, ***qnrB***, *qnrA*, *qnrD*	I	1 (33)
		***gyrA* S83F, S83Y, D87N, D87Y, D87A, D87G, D87V**	I	1 (33, 34)
		***gyrB* S464F, S464Y, Q465R, Q465L**	I	1 (33)
		***parC* S80I, E84G, E84K**	I	1 (33)
		***parE* L416F, D420N**	I	1 (33, 35, 36)
Sulfonamides	Sulfamethoxazole (SXM)	***sul1***, ***sul2***	R	1 (32)
Folate Pathway Inhibitors	Trimethoprim (TMP)	***dfrA1***, ***dfrA5***, ***dfrA7***, ***drfA14***, ***dfrA15***, ***dfrA17***, ***dfrA18***	R	1 (32)
	Tetracycline (TCY)	***tetA*(A)**, ***tetA*(B)**, ***tetA*(C)**, ***tetA*(D)**	R	1 (31)
Macrolides	Azithromycin (AZM)	*ermA*, *ermB*, *ermC*	R	1
		ereA, *ereB*	R	1
		mefA	R	1
		mphA, *mphB*	R	1
		msrA, *msrD*	R	1
		***acrB* R717Q**	R	1 (12)
Penicillins	Ampicillin (AMP)	***bla***$_{\textbf{TEM-1}}$	R	1 (31)
		bla$_{\text{OXA-1}}$, *bla*$_{\text{OXA-7}}$	R	1
		Determinants of CEP and MEM	R	2,3
Cephalosporins	Extended-spectrum cephalosporins (CEP)	*bla*$_{\text{CMY-2}}$	R	1
		ampC	R	1
		bla$_{\textbf{CTX-M}}$	R	2 (37)
		bla$_{\textbf{SHV}}$	R	2 (38)
		bla$_{\text{OXA-11}}$, *bla*$_{\text{OXA-15}}$	R	2
		bla$_{\text{TEM-10}}$, *bla*$_{\text{TEM-124}}$	R	2
		Determinants of MEM	R	3
Carbapenems	Meropenem (MEM)	*bla*$_{\text{AIM}}$	R	3
		bla$_{\text{BIC}}$	R	3
		bla$_{\text{DIM}}$	R	3
		bla$_{\text{GES}}$	R	3
		bla$_{\text{GIM}}$	R	3
		bla$_{\text{IMI}}$	R	3
		bla$_{\text{IMP}}$	R	3
		bla$_{\text{KPC}}$	R	3
		bla$_{\text{LMB}}$	R	3

		bla$_{NDM}$	R	3
		bla$_{NMCA}$	R	3
		bla$_{OXA-48}$ like	R	3
		bla$_{SIM}$	R	3
		bla$_{SPM}$	R	3
		bla$_{VIM}$	R	3
Polymixins	Colistin (CST)	*mcr-12345678*	R	4

Supplementary Table 3. Distribution of known fluoroquinolone (Flq) resistance determinants (genes and mutations) in the 4389 public genomes in Pathogenwatch, and the linked genomic prediction of ciprofloxacin resistance provided by in the Antibiotics table.

No. of Genomes	No. of Flq resistance genes	No. of Flq resistance mutations	Flq resistance determinants identified in the genomes	Genomic prediction of ciprofloxacin resistance
1939	0	0	None	Susceptible
1219	0	1	*gyrA*_S83F	Intermediate
421	0	1	*gyrA*_S83Y	Intermediate
80	0	1	*gyrB*_S464F	Intermediate
68	0	1	*gyrA*_D87N	Intermediate
26	0	1	*gyrA*_D87G	Intermediate
16	0	1	*gyrB*_S464Y	Intermediate
14	0	1	*gyrA*_D87Y	Intermediate
1	0	1	*gyrA*_D87V	Intermediate
1	0	1	*gyrA*_D87A	Intermediate
1	0	1	*gyrB*_Q465L	Intermediate
158	0	2	*gyrA*_S83F,*parE*_D420N	Intermediate
27	0	2	*gyrA*_S83F,*parC*_E84G	Intermediate
23	0	2	*gyrA*_S83F,*parE*_L416F	Intermediate
7	0	2	*gyrA*_S83F,*parC*_E84K	Intermediate
4	0	2	*gyrA*_S83F,*parC*_S80I	Intermediate
3	0	2	*gyrA*_S83Y,*parE*_D420N	Intermediate
1	0	2	*gyrB*_S464F,*gyrB*_Q465L	Intermediate
1	0	2	*gyrA*_D87N,*gyrB*_Q465L	Intermediate
192	0	3	*gyrA*_S83F,*gyrA*_D87N,*parC*_S80I	Resistant
9	0	3	*gyrA*_S83F,*gyrA*_D87G,*parC*_E84K	Intermediate
6	0	3	*gyrA*_S83F,*gyrB*_Q465R,*parE*_D420N	Intermediate
5	0	3	*gyrA*_S83F,*gyrA*_D87V,*parC*_S80I	Intermediate
2	0	3	*gyrA*_S83F,*gyrA*_D87G,*parC*_S80I	Intermediate
1	0	3	*gyrA*_S83F,*gyrA*_D87N,*parC*_E84K	Intermediate
1	0	3	*gyrA*_S83Y,*gyrA*_D87G,*parC*_S80I	Intermediate
1	0	3	*gyrA*_S83F,*gyrA*_D87G,*parE*_D420N	Intermediate
1	0	3	*gyrA*_S83F,*gyrA*_D87G,*parC*_E84G	Intermediate
3	1	0	*qnrS*	Intermediate
87	1	1	*qnrS*,*gyrA*_S83F	Resistant
64	1	1	*qnrS*,*gyrA*_S83Y	Resistant
2	1	1	*qnrB*,*gyrA*_S83F	Resistant
2	1	2	*qnrB*,*gyrA*_S83F,*parC*_E84K	Resistant
3	1	3	*qnrB*,*gyrA*_S83F,*gyrA*_D87N,*parC*_S80I	Resistant

Supplementary Table 4. Reference genomes used to compute the Pathogenwatch *S.* Typhi core genome library (N=26) and the population tree (N=19).

Strain Name	Country	Collection Year	MLST ST	GenoTyphi genotype	Assembly Length	N50	No. contigs	Non-ATCG	% GC Content	% Core Families	Accessions	Study Accession	PMID	Core Library	Population Tree Reference
E02-1180	India	2002	2	0.0.3	4838914	4793071	4	0	52.1	100	ERR581073, ERR581085	PRJEB5919	25961941	yes	yes
E00-7866	Morocco	2000	2	0.1	4831122	4797408	3	0	52.1	100	ERR581072, ERR581087	PRJEB5919	25961941	yes	yes
76-1292	Democratic Republic of Congo	1976	8	1.1.3	4934685	4780549	3	0	52.1	100	ERR601541, ERR601549	PRJEB5919	25961941	yes	yes
M326	Unknown	1939	2	1.2.1	4775555	204340	47	8	52	100	ERR279243	PRJEB3255	26411565	no	yes
11909_3	Mexico	2011	2	2.0.2	4811824	4753305	4	0	52	100	ERR584382, ERR584388	PRJEB5919	25961941	yes	yes
E98-3139	Mexico	1998	2	2.0.2	4675865	3003486	6	0	52.1	100	ERR581098, ERR581108, ERR601543	PRJEB5919	25961941	yes	no
ERL024120	Indonesia	2002	2138	2.1.2	4811886	4811886	1	0	52.1	100	ERR581076, ERR581091	PRJEB5919	25961941	yes	yes
M223	Unknown	1939	2	2.1.7	4849011	4806333	3	0	52.1	100	ERR581077, ERR581088	PRJEB5919	25961941	yes	no
80-2002	Madagascar	1980	2	2.2	5061131	4819925	4	0	52.1	100	ERR601542, ERR601550	PRJEB5919	25961941	yes	no
ERL114000	Nepal	2011	2	2.2	4776059	4776059	1	0	52.1	100	ERR581075, ERR581086	PRJEB5919	25961941	yes	no
UI4692	Laos	2004	2	2.2.2,2.2.3	4707798	185395	43	0	52.1	100	ERR331348	PRJEB3215	25961941	no	yes
ERL103914	South America	2010	2	2.3.2	4789623	4789623	1	0	52.1	100	ERR581074, ERR581092	PRJEB5919	25961941	yes	no
ST1921-06	Argentina	2006	2	2.3.3	4718006	204238	42	0	52.1	100	ERR353335	PRJEB3215	25961941	no	yes
LNT1360	Laos	2010	2218	2.4	4794285	206322	40	2	52.1	100	ERR326664	PRJEB3215	25961941	yes	no
D50739	Malawi	2009	2	2.4.1	4704071	206289	45	24	52.1	100	ERR279161	PRJEB3215	25961941	yes	yes
E98-0664	Kenya	1998	2	2.5	4764488	4752533	3	0	52.1	100	ERR601554, ERR601551	PRJEB5919	25961941	yes	yes
H12ESR00755-001A	Philippines	2012	1	3	4814227	4802146	2	0	52.1	100	ERR581080, ERR581089	PRJEB5919	25961941	yes	yes
Quailes	USA	1958	2	3.1	4841927	4797293	4	0	52.1	100	ERR634098, ERR634100, ERR654508	PRJEB5919	25961941	yes	no
404Ty	Indonesia	1983	2	3.1.2	4890679	4807711	6	0	52	100	ERR581078, ERR581090	PRJEB5919	25961941	yes	yes
CT18	Vietnam	1993	2	3.2.1	5133713	4809037	3	0	51.9	100	GCA_000195995.1	PRJNA236	11677608	yes	yes

ERL041834	India	2004	2	3.3	4892436	4675147	5	0	52.1	100	ERR581081, ERR581083	PRJEB5919	25961941	yes	yes
H12ESR04734-001A	India	2012	2	3.3	4943859	4876386	5	0	52.1	100	ERR581099, ERR581105	PRJEB5919	25961941	yes	no
002168	Cambodia	2010	1	3.4	4764266	217780	42	2	52.1	100	ERR360747	PRJEB3215	25961941	yes	no
LNT1148	Laos	2001	1	3.4	4743503	204320	46	2	52.1	100	ERR340779	PRJEB3215	25961941	yes	yes
ERL024919	Samoa	2002	1	3.5.4	4805266	4784637	2	0	52.1	100	ERR581094, ERR581102	PRJEB5919	25961941	yes	no
H12ESR00394-001A	Samoa	2012	1	3.5.4	4808147	4808147	1	0	52.1	100	ERR581095, ERR581103	PRJEB5919	25961941	yes	yes
Ty2	Russia	1916	1	4.1	4791961	4791961	1	11	52.1	100	GCA_000007545.1	PRJNA371	12644504	yes	yes
ERL072973	Fiji	2007	1	4.2.2	4806043	4789877	2	0	52.1	100	ERR581097, ERR581107	PRJEB5919	25961941	yes	yes
007898	Cambodia	2010	1	4.3.1.1	4793925	4793925	1	0	52.1	100	ERR752439, ERR752446	PRJEB5919	25961941	yes	yes

Supplementary References

1. Ahmad N, Hii SY, Hashim R, Issa R. Draft Genome Sequence of *Salmonella enterica* Serovar Typhi IMR_TP298/15, a Strain with Intermediate Susceptibility to Ciprofloxacin, Isolated from a Typhoid Outbreak. Genome Announc. 2017;5(9).
2. Ashton PM, Nair S, Peters TM, Bale JA, Powell DG, Painset A, et al. Identification of *Salmonella* for public health surveillance using whole genome sequencing. PeerJ. 2016;4:e1752.
3. Baker KS, Burnett E, McGregor H, Deheer-Graham A, Boinett C, Langridge GC, et al. The Murray collection of pre-antibiotic era Enterobacteriacae: a unique research resource. Genome Med. 2015;7:97.
4. Britto CD, Dyson ZA, Duchene S, Carter MJ, Gurung M, Kelly DF, et al. Laboratory and molecular surveillance of paediatric typhoidal *Salmonella* in Nepal: Antimicrobial resistance and implications for vaccine policy. PLoS Negl Trop Dis. 2018;12(4):e0006408.
5. Britto CD, Dyson ZA, Mathias S, Bosco A, Dougan G, Jose S, et al. Persistent circulation of a fluoroquinolone-resistant *Salmonella enterica* Typhi clone in the Indian subcontinent. J Antimicrob Chemother. 2020;75(2):337-41.
6. Burnsed LJ, Kovar LD, Angelo KM, Trees EK, Concepcion-Acevedo J, McDermott MD, et al. Use of whole genome sequencing to complement characterisation of a typhoid fever outbreak among a Marshallese community: Oklahoma, 2015. Epidemiol Infect. 2018:1-7.
7. Djeghout B, Saha S, Sajib MSI, Tanmoy AM, Islam M, Kay GL, et al. Ceftriaxone-resistant *Salmonella* Typhi carries an IncI1-ST31 plasmid encoding CTX-M-15. J Med Microbiol. 2018;67(5):620-7.
8. Dyson ZA, Thanh DP, Bodhidatta L, Mason CJ, Srijan A, Rabaa MA, et al. Whole Genome Sequence Analysis of *Salmonella* Typhi Isolated in Thailand before and after the Introduction of a National Immunization Program. PLoS Negl Trop Dis. 2017;11(1):e0005274.
9. Gul D, Potter RF, Riaz H, Ashraf ST, Wallace MA, Munir T, et al. Draft Genome Sequence of a *Salmonella enterica* Serovar Typhi Strain Resistant to Fourth-Generation Cephalosporin and Fluoroquinolone Antibiotics. Genome Announc. 2017;5(42).
10. Hendriksen RS, Leekitcharoenphon P, Lukjancenko O, Lukwesa-Musyani C, Tambatamba B, Mwaba J, et al. Genomic signature of multidrug-resistant *Salmonella enterica* serovar typhi isolates related to a massive outbreak in Zambia between 2010 and 2012. J Clin Microbiol. 2015;53(1):262-72.
11. Hendriksen RS, Leekitcharoenphon P, Mikoleit M, Jensen JD, Kaas RS, Roer L, et al. Genomic dissection of travel-associated extended-spectrum-beta-lactamase-producing *Salmonella enterica* serovar Typhi isolates originating from the Philippines: a one-off occurrence or a threat to effective treatment of typhoid fever? J Clin Microbiol. 2015;53(2):677-80.
12. Hooda Y, Sajib MSI, Rahman H, Luby SP, Bondy-Denomy J, Santosham M, et al. Molecular mechanism of azithromycin resistance among typhoidal *Salmonella* strains in Bangladesh identified through passive pediatric surveillance. PLoS Negl Trop Dis. 2019;13(11):e0007868.
13. Ingle DJ, Nair S, Hartman H, Ashton PM, Dyson ZA, Day M, et al. Informal genomic surveillance of regional distribution of *Salmonella* Typhi genotypes and antimicrobial resistance via returning travellers. PLoS Negl Trop Dis. 2019;13(9):e0007620.
14. Klemm EJ, Shakoor S, Page AJ, Qamar FN, Judge K, Saeed DK, et al. Emergence of an Extensively Drug-Resistant *Salmonella enterica* Serovar Typhi Clone Harboring a Promiscuous Plasmid Encoding Resistance to Fluoroquinolones and Third-Generation Cephalosporins. mBio. 2018;9(1).
15. Matono T, Morita M, Yahara K, Lee KI, Izumiya H, Kaku M, et al. Emergence of Resistance Mutations in *Salmonella enterica* Serovar Typhi Against Fluoroquinolones. Open Forum Infect Dis. 2017;4(4):ofx230.
16. Oo KM, Myat TO, Htike WW, Biswas A, Hannaway RF, Murdoch DR, et al. Molecular mechanisms of antimicrobial resistance and phylogenetic relationships of *Salmonella enterica* isolates from febrile patients in Yangon, Myanmar. Trans R Soc Trop Med Hyg. 2019;113(10):641-8.
17. Park SE, Pham DT, Boinett C, Wong VK, Pak GD, Panzner U, et al. The phylogeography and incidence of multi-drug resistant typhoid fever in sub-Saharan Africa. Nat Commun. 2018;9(1):5094.
18. Pham Thanh D, Karkey A, Dongol S, Ho Thi N, Thompson CN, Rabaa MA, et al. A novel ciprofloxacin-resistant subclade of H58 Salmonella Typhi is associated with fluoroquinolone treatment failure. Elife. 2016;5:e14003.
19. Phoba MF, Barbe B, Lunguya O, Masendu L, Lulengwa D, Dougan G, et al. Salmonella enterica serovar Typhi Producing CTX-M-15 Extended Spectrum beta-Lactamase in the Democratic Republic of the Congo. Clin Infect Dis. 2017;65(7):1229-31.
20. Pragasam AK, Pickard D, Wong V, Dougan G, Kang G, Thompson A, et al. Phylogenetic Analysis Indicates a Longer Term Presence of the Globally Distributed H58 Haplotype of *Salmonella* Typhi in Southern India. Clin Infect Dis. 2020.

21. Rodrigues C, Kapil A, Sharma A, Devanga Ragupathi NK, Inbanathan FY, Veeraraghavan B, et al. Whole-Genome Shotgun Sequencing of Cephalosporin-Resistant *Salmonella enterica* Serovar Typhi. Genome Announc. 2017;5(10).
22. Sah R, Donovan S, Seth-Smith HMB, Bloemberg G, Wuthrich D, Stephan R, et al. A novel lineage of ceftriaxone-resistant *Salmonella* Typhi from India that is closely related to XDR S. Typhi found in Pakistan. Clin Infect Dis. 2019.
23. Tanmoy AM, Westeel E, De Bruyne K, Goris J, Rajoharison A, Sajib MSI, et al. *Salmonella enterica* Serovar Typhi in Bangladesh: Exploration of Genomic Diversity and Antimicrobial Resistance. mBio. 2018;9(6).
24. Wong VK, Baker S, Pickard DJ, Parkhill J, Page AJ, Feasey NA, et al. Phylogeographical analysis of the dominant multidrug-resistant H58 clade of *Salmonella* Typhi identifies inter- and intracontinental transmission events. Nat Genet. 2015;47(6):632-9.
25. Wong VK, Baker S, Connor TR, Pickard D, Page AJ, Dave J, et al. An extended genotyping framework for *Salmonella enterica* serovar Typhi, the cause of human typhoid. Nat Commun. 2016;7:12827.
26. Wong VK, Holt KE, Okoro C, Baker S, Pickard DJ, Marks F, et al. Molecular Surveillance Identifies Multiple Transmissions of Typhoid in West Africa. PLoS Negl Trop Dis. 2016;10(9):e0004781.
27. Pathogenwatch S. Typhi AMR library. https://gitlab.com/cgps/pathogenwatch/amr-libraries/-/blob/master/90370.toml. Accessed 28 Jan 2021.
28. Pathogenwatch ESBL library. https://gitlab.com/cgps/pathogenwatch/amr-libraries/-/blob/master/gram_neg_esbl.toml. Accessed 28 Jan 2021.
29. Pathogenwatch carbapenemases library. https://gitlab.com/cgps/pathogenwatch/amr-libraries/-/blob/master/gram_neg_carbapenemases.toml. Accessed 28 Jan 2021.
30. Pahogenwatch colistin resistance library. https://gitlab.com/cgps/pathogenwatch/amr-libraries/-/blob/master/gram_neg_colistin.toml. Accessed 28 Jan 2021.
31. Britto CD, Wong VK, Dougan G, Pollard AJ. A systematic review of antimicrobial resistance in *Salmonella enterica* serovar Typhi, the etiological agent of typhoid. PLoS Negl Trop Dis. 2018;12(10):e0006779.
32. Crump JA, Sjolund-Karlsson M, Gordon MA, Parry CM. Epidemiology, Clinical Presentation, Laboratory Diagnosis, Antimicrobial Resistance, and Antimicrobial Management of Invasive *Salmonella* Infections. Clin Microbiol Rev. 2015;28(4):901-37.
33. Cuypers WL, Jacobs J, Wong V, Klemm EJ, Deborggraeve S, Van Puyvelde S. Fluoroquinolone resistance in *Salmonella*: insights by whole-genome sequencing. Microb Genom. 2018;4(7).
34. Roumagnac P, Weill FX, Dolecek C, Baker S, Brisse S, Chinh NT, et al. Evolutionary history of Salmonella typhi. Science. 2006;314(5803):1301-4.
35. Accou-Demartin M, Gaborieau V, Song Y, Roumagnac P, Marchou B, Achtman M, et al. *Salmonella enterica* Serotype Typhi with nonclassical quinolone resistance phenotype. Emerg Infect Dis. 2011;17(6):1091-4.
36. Song Y, Roumagnac P, Weill FX, Wain J, Dolecek C, Mazzoni CJ, et al. A multiplex single nucleotide polymorphism typing assay for detecting mutations that result in decreased fluoroquinolone susceptibility in *Salmonella enterica* serovars Typhi and Paratyphi A. J Antimicrob Chemother. 2010;65(8):1631-41.
37. Al Naiemi N, Zwart B, Rijnsburger MC, Roosendaal R, Debets-Ossenkopp YJ, Mulder JA, et al. Extended-spectrum-beta-lactamase production in a *Salmonella enterica* serotype Typhi strain from the Philippines. J Clin Microbiol. 2008;46(8):2794-5.
38. Ahmed D, Hoque A, Mazumder R, Nahar K, Islam N, Gazi SA, et al. *Salmonella enterica* serovar Typhi strain producing extended-spectrum beta-lactamases in Dhaka, Bangladesh. J Med Microbiol. 2012;61(Pt 7):1032-3.
