# Supplementary material for: A global resource for genomic predictions of antimicrobial resistance and surveillance of Salmonella Typhi at pathogenwatch
Source: Nat Commun. 2021 May 17;12:2879. doi: 10.1038/s41467-021-23091-2 (PMC8128892; doi:10.1038/s41467-021-23091-2)
Supplement: Supplementary file 3 — Descriptions of Additional Supplementary Files [file 41467_2021_23091_MOESM3_ESM.pdf]

Descriptions of Additional Supplementary Files

Supplementary Data 1
Description: Metadata, assembly stats, typing information and species identification of the S. Typhi public genomes available on Pathogenwatch.

Supplementary Data 2
Description: Metadata and accessions of 118 S. Typhi genomes comprising tree benchmarking Dataset I.

Supplementary Data 3
Description: Metadata and accessions of 138 S. Typhi genomes comprising tree benchmarking Dataset II.

Supplementary Data 4
Description: Metadata and accessions of 43 S. Typhi genomes comprising tree benchmarking Dataset III.
