# Supplementary material for: A global resource for genomic predictions of antimicrobial resistance and surveillance of Salmonella Typhi at pathogenwatch
Source: Nat Commun. 2021 May 17;12:2879. doi: 10.1038/s41467-021-23091-2 (PMC8128892; doi:10.1038/s41467-021-23091-2)
Supplement: Supplementary file 8 — Reporting Summary [file 41467_2021_23091_MOESM8_ESM.pdf]

nature research

Corresponding author(s): Silvia Argimón and David M. Aanensen

Last updated by author(s): Apr 9, 2021

Reporting Summary

Nature Research wishes to improve the reproducibility of the work that we publish. This form provides structure for consistency and transparency in reporting. For further information on Nature Research policies, see our Editorial Policies and the Editorial Policy Checklist.

Statistics

For all statistical analyses, confirm that the following items are present in the figure legend, table legend, main text, or Methods section.

n/a	Confirmed	
☐	☒	The exact sample size (n) for each experimental group/condition, given as a discrete number and unit of measurement
☒	☐	A statement on whether measurements were taken from distinct samples or whether the same sample was measured repeatedly
☒	☐	The statistical test(s) used AND whether they are one- or two-sided<br>*Only common tests should be described solely by name; describe more complex techniques in the Methods section.*
☒	☐	A description of all covariates tested
☒	☐	A description of any assumptions or corrections, such as tests of normality and adjustment for multiple comparisons
☒	☐	A full description of the statistical parameters including central tendency (e.g. means) or other basic estimates (e.g. regression coefficient) AND variation (e.g. standard deviation) or associated estimates of uncertainty (e.g. confidence intervals)
☒	☐	For null hypothesis testing, the test statistic (e.g. F, t, r) with confidence intervals, effect sizes, degrees of freedom and P value noted<br>*Give P values as exact values whenever suitable.*
☒	☐	For Bayesian analysis, information on the choice of priors and Markov chain Monte Carlo settings
☒	☐	For hierarchical and complex designs, identification of the appropriate level for tests and full reporting of outcomes
☒	☐	Estimates of effect sizes (e.g. Cohen's d, Pearson's r), indicating how they were calculated

Our web collection on statistics for biologists contains articles on many of the points above.

Software and code

Policy information about availability of computer code

Data collection	No commercial, open-source or custom code was used to collect the data (genomes or linked metadata) in this study.

Data analysis	Ariba v.2.14.4<br>BLASTn v2.2.31+<br>Dendroscope v3.5<br>epiR v1.0-14<br>FastTree v2.1.8<br>MAFFT v7.2.2.0<br>Pathogenwatch (https://pathogen.watch/)<br>RAxML v8.2.8<br>Resfinder v3.2.1<br>Roary v3.6.0 and v 3.2.0<br>SPAdes v3.9.0<br>Tableau Desktop v2018.3.11<br>Treescape v1.10.18<br>Velvet v1.2<br>VelvetOptimizer v2.2.5<br>Material Design Lite v1.3.0<br>Leaflet v1.4.0<br>Sigma v1.2.1<br>Mash v2.1<br>AMR benchmarking script https://gitlab.com/cgps/pathogenwatch/publications/-/tree/master/styphi

For manuscripts utilizing custom algorithms or software that are central to the research but not yet described in published literature, software must be made available to editors and reviewers. We strongly encourage code deposition in a community repository (e.g. GitHub). See the Nature Research guidelines for submitting code & software for further information.

Data

Policy information about availability of data

All manuscripts must include a data availability statement. This statement should provide the following information, where applicable:

- Accession codes, unique identifiers, or web links for publicly available datasets
- A list of figures that have associated raw data
- A description of any restrictions on data availability

The genome assemblies and linked metadata analysed in this study are available from: https://pathogen.watch/collection/07lsscrbhu2x-public-genomes, https://pathogen.watch/collection/g5pbucot6e58-hendriksen-et-al-2015, and https://pathogen.watch/collection/11lsok8nrzts-wong-et-al-2018-idcases-15e00492
The raw sequence data is available from the European Nucleotide Archive via the accessions provided in Supplementary Table 4, and also found in the Metadata Table of https://pathogen.watch/collection/07lsscrbhu2x-public-genomes.

Field-specific reporting

Please select the one below that is the best fit for your research. If you are not sure, read the appropriate sections before making your selection.

☒ Life sciences ☐ Behavioural & social sciences ☐ Ecological, evolutionary & environmental sciences

For a reference copy of the document with all sections, see nature.com/documents/nr-reporting-summary-flat.pdf

Life sciences study design

All studies must disclose on these points even when the disclosure is negative.

Sample size	No sample size power calculation was carried out as we included all public and published genomes that passed quality control.
Data exclusions	Genomes were excluded when the assemblies did not pass quality control as per pre-established criteria (number of contigs, assembly length, N50, non-ATCG characters, GC content, number of core matches). Genomes were excluded from the Pathogenwatch application if the assemblies either contained more than 700 contigs, more than 50,000 non-ATCG characters, a GC content below the smallest GC content or above than the largest GC content of the S. enterica subsp enterica genomes in RefSeq, or a total length that is <10% smaller than the smallest genome or >10% larger than the largest S. enterica subsp enterica genome in RefSeq,
Replication	The reproducibility of the results was ensured by the quality control of the sequence data, the inclusion of bootstrap replication in the inference of phylogenetic trees (500 replicates) when appropriate, and by conducting independent analyses with alternative methods when necessary. The Pathogenwatch website allows samples to be run through a reproducible pipeline.
Randomization	Random sampling did not apply to our study, as the genomes included in this study are all those public and published that passed quality control as described in the Methods and we did not make comparisons between a treatment and a control group.
Blinding	Blinding was not applicable to our study, as it did not include experimental and control groups.

Reporting for specific materials, systems and methods

We require information from authors about some types of materials, experimental systems and methods used in many studies. Here, indicate whether each material, system or method listed is relevant to your study. If you are not sure if a list item applies to your research, read the appropriate section before selecting a response.

Materials & experimental systems

n/a	Involved in the study
☒	☐ Antibodies
☒	☐ Eukaryotic cell lines
☒	☐ Palaeontology and archaeology
☒	☐ Animals and other organisms
☒	☐ Human research participants
☒	☐ Clinical data
☒	☐ Dual use research of concern

Methods

n/a	Involved in the study
☒	☐ ChIP-seq
☒	☐ Flow cytometry
☒	☐ MRI-based neuroimaging
